# Surgical Treatment of Intrapelvic Pseudotumour after Hip Resurfacing Arthroplasty: Case Report and Literature Review

**DOI:** 10.1155/2018/3808362

**Published:** 2018-10-25

**Authors:** Cristian Barrientos, Julian Brañes, José-Luis Llanos, Alvaro Martinez, Maximiliano Barahona

**Affiliations:** ^1^Orthopaedic Department at Hospital Clinico Universidad de Chile, Santos Dumontt 999, Santiago, Chile; ^2^Hospital Clinico Universidad de Chile, 999 Santos Dumont av., Independencia, Santiago 8380456, Chile; ^3^Orthopaedic Department at Hospital San José, 1196 San Jose av. Independencia, Santiago 8380419, Chile; ^4^Coloproctology Surgery at Hospital Clinico Universidad de Chile, Santos Dumontt 999, Santiago, Chile

## Abstract

Hip replacement is the surgery of the last century due to its impact on the quality of life. A pseudotumour is a rare complication of hip arthroplasty, and it is related to a metal-bearing surface. Pseudotumour is a challenging scenario for hip surgeons due to poor clinical outcomes. The patient consulted for hip pain and paresthesia in the left lower extremity, and analyses showed that the cause was a sizeable intrapelvic pseudotumour. A multidisciplinary team surgery was planned. At first, an infraumbilical approach was made to resect the intrapelvic-retroperitoneum portion of the pseudotumour. Then, a posterolateral hip approach was performed, to resect the remaining portion of the pseudotumour and revision arthroplasty. At five years of follow-up, there are no clinical or imaging signs of recurrence of the pseudotumour. Treatment evidence is limited to a series of cases and expert opinions; we encourage complete resection and revision arthroplasty.

## 1. Introduction

Hip replacement is the surgery of the last century due to its impact on the life of quality [1]. Different types of prosthesis design exist. In traditional total hip replacement (THR), the head of the femur is removed, and a stem is placed inside the femoral metaphysis. In the early 1990s, hip resurfacing arthroplasty (HRA) was introduced; there was no significant difference in the acetabulum component, but in the femoral side, only the femur head is replaced and a short stem is used; therefore, the metaphysis of the femur remains intact. So, compared to THR, HRA preserves more bone, has lower stress shielding, and has lower surgery morbidity (i.e., less bleeding). A few case series have shown good results in the short and medium terms [2, 3].

Two major concerns for HRA are described: a higher incidence of neck fracture because, for design, more stress is placed in the neck of the femur and the other concern is metal ion risk [4]. All prosthesis models have a bearing surface: one in the acetabulum and other in the femoral head. Different materials have been used as a bearing surface, such as polyethylene, ceramic, and metal. The amount and size of the debris depend on the material used as a bearing surface and are associated with the aseptic loosening of the prosthesis [5]. HRA uses metal on metal-bearing surface only, and this bearing surface is related to a particular complication known as a pseudotumour.

The most specific histological finding of a pseudotumor is a lymphocytic immune response described as an aseptic lymphocytic injury-associated vasculitis (ALVAL) [6]. Its formation has been associated with high serum levels of cobalt and chromium; however, the physiopathology remains unclear given that the most frequent findings consist of an infiltrate of monocytes, reparative tissue, giant cells, and areas of necrosis [7, 8].

The surgical treatment of pseudotumour is a theme of discussion, and only a few clinical series have been published [9]. Treatment should include pseudotumour resection and revision hip arthroplasty (RHA). The extension of the pseudotumour resection is on a debate and, sometimes due to its extension, is not completely resected. RHA consists of removing all the prosthetic components, including the bearing surface and replacing them with a new prosthesis [10].

Our purpose is to report the case of a patient in which a complete resection of an intrapelvic pseudotumor that compromised the retroperitoneum, following an HRA, was performed, showing no signs of recurrence after five years of follow-up.

## 2. Case Report

A 46-year-old female patient underwent a left hip resurfacing arthroplasty (Birmingham®, UK) for severe hip osteoarthritis, secondary to developmental hip dysplasia, in 2005. She had a good initial outcome with no complications. The surgery was performed in another centre.

**Figure 1:**
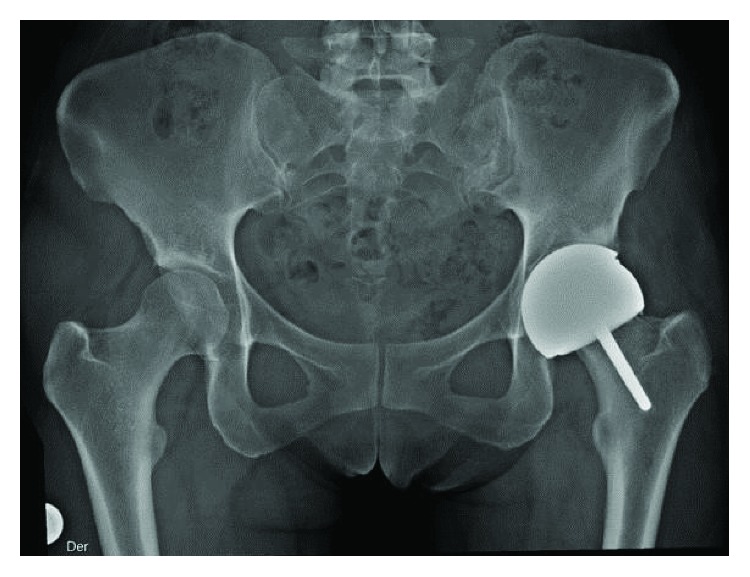
Postoperative AP radiograph showing the left hip's resurfacing arthroplasty. Femoral head was 46 mm. Acetabular cup was 52 mm and was placed too vertical; malposition of the components is a risk factor for pseudotumour [8].

She consulted with us for the first time six years later (2011) complaining about hip pain and paresthesia in the anterior left thigh, which progressively compromised her function. She denied fever or other signs of infection. Physical exams revealed a mild claudication gait and limited active and passive hip flexion. No palpable masses or skin lesions were observed. Laboratory analyses showed a WBC, ESR, and CRP within normal limits. No signs of osteolysis were found in the hip anteroposterior radiography; nevertheless, a vertical cup was noted (Figure 1). It was compared with the immediate postsurgery radiography, and no change was noted.

**Figure 2:**
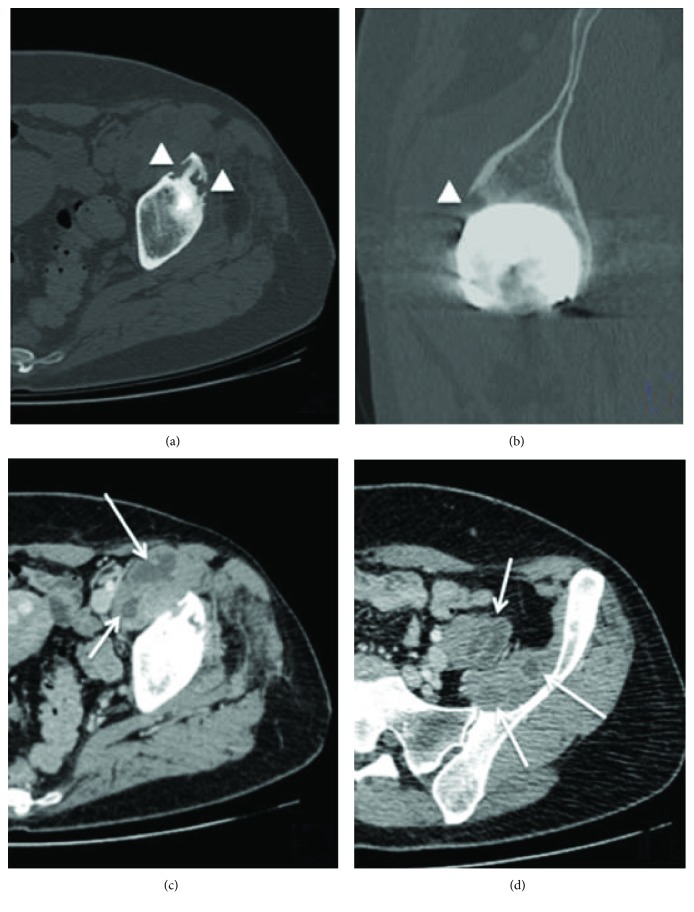
Computed tomography of the left hip: axial (a) and sagittal (b) views show periprosthetic osteolytic lesions (arrow head) in the anterior acetabular roof. Axial views (c, d) show a polylobate cystic lesion in the retroperitoneum compromising the left iliopsoas muscle with white arrows.

**Figure 3:**
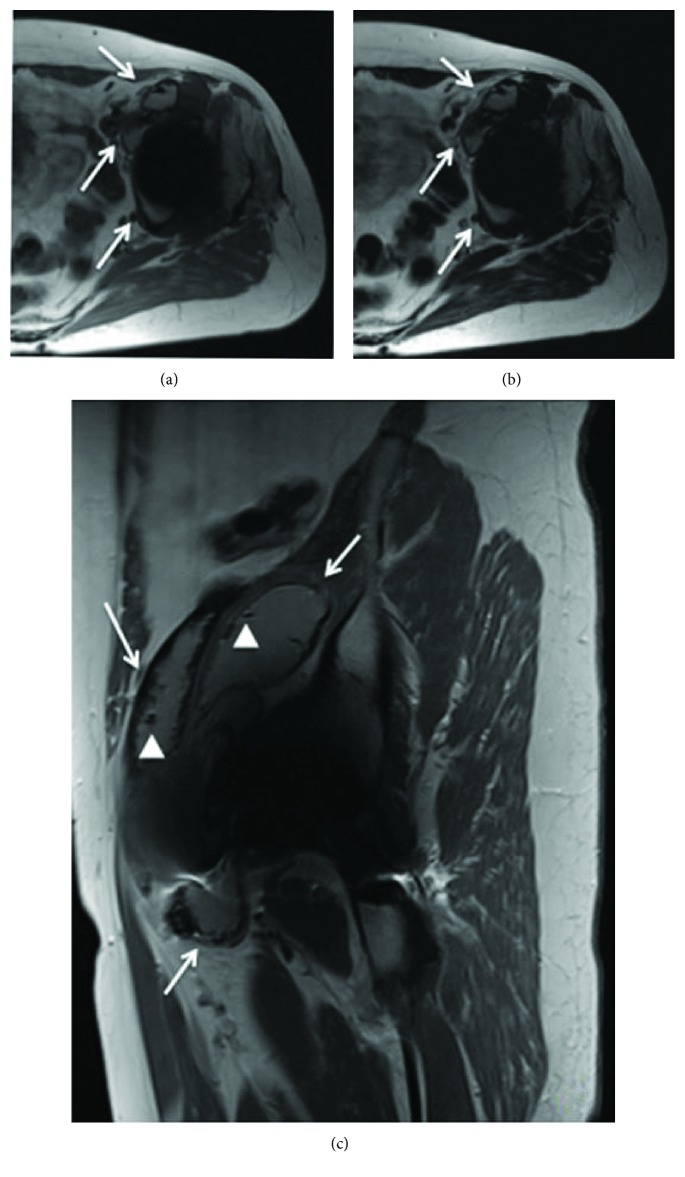
In the metal artefact reduction sequence magnetic resonance imaging (MARS MRI), axial T1 (a), axial T2 (b), and sagittal T1 (c) show the presence of two lesions of thick walls located anterior to the psoas and iliacus (arrows) muscle. Within the lesion, a small focus of artefact is seen (arrow heads). This finding is critical to determining that the aetiology of the pseudotumour is “metallosis.”

Computed tomography and an MRI demonstrated a biloculate hypodense mass of approximately 34 × 19 cm that extended from the retroperitoneum, compromising the left iliopsoas muscle and an intimate contact with the femoral vessels, to the left hip and the left femoral-cutaneous nerve (Figures 2 and 3). A routine hip arthrocentesis was performed to rule out infection. The cytochemical and Gram analyses were negative for infection. Cultures were negative after 14 days.

Thus, a pseudotumour was the diagnosis, and surgery led by an orthopaedic surgeon and a coloproctology surgeon was planned. The aim was to remove the pseudotumor entirely and to perform an RHA. As the CT shows (Figure 2(d)), the intrapelvic mass was significant, so it was decided to start with a laparotomy by the coloproctology surgeon.

**Figure 4:**
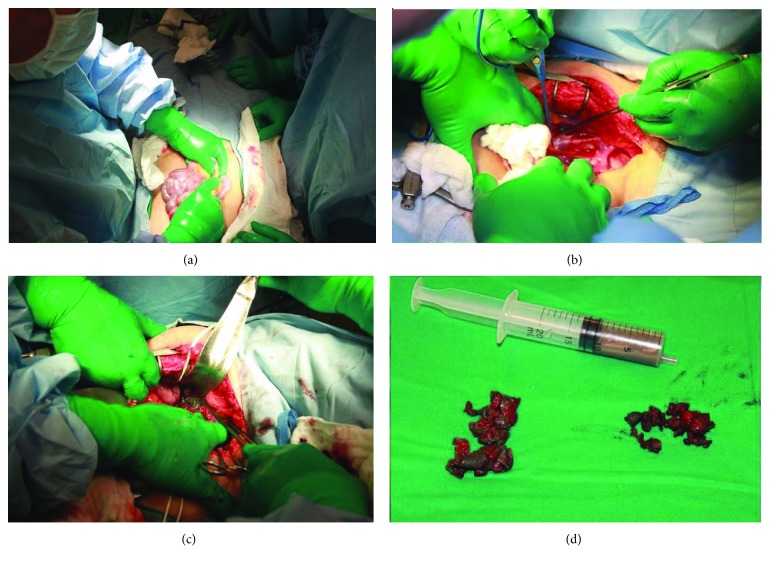
Four images from the first step of the surgery are shown. After a midline longitudinal infraumbilical approach, the left paracolic gutter was addressed (a) to obtain access to the retroperitoneum (b); a cystic tumour was observed in intimate contact with the iliopsoas muscle and the femoral nerve and vessels (c). The pseudotumour was punctured, and a grey milky-like fluid was obtained; then, the tumour was completely resected (d).

First, the patient was positioned supine, and an infraumbilical laparotomy was performed; the left paracolic gutter was dissected to address the retroperitoneum. The iliac vessels and the left ureter were protected, and an irregular cystic mass was observed in direct contact with the psoas muscle and femoral bundle. It was punctured, and an abundant grey milky-like fluid was obtained. The membrane of the cyst was carefully removed, protecting the vessels and the femoral nerve. The lesion was completely resected, and samples were sent for biopsy and cultures (Figure 4).

**Figure 5:**
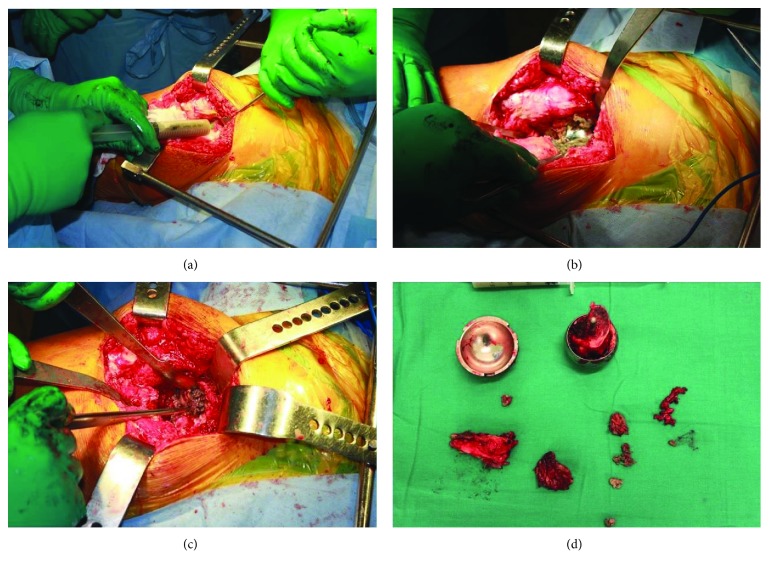
Four images from the second step of the surgery are shown. Through a posterolateral hip approach, an RHA was performed. The capsule was looking distend, so it was punctured (a), after capsulotomy the pseudotumour was evident (b), all components were removed, and an anterosuperior acetabulum wall defect was observed (c). Tumour sample, liquid and the hip prosthesis removed are shown in (d).

**Figure 6:**
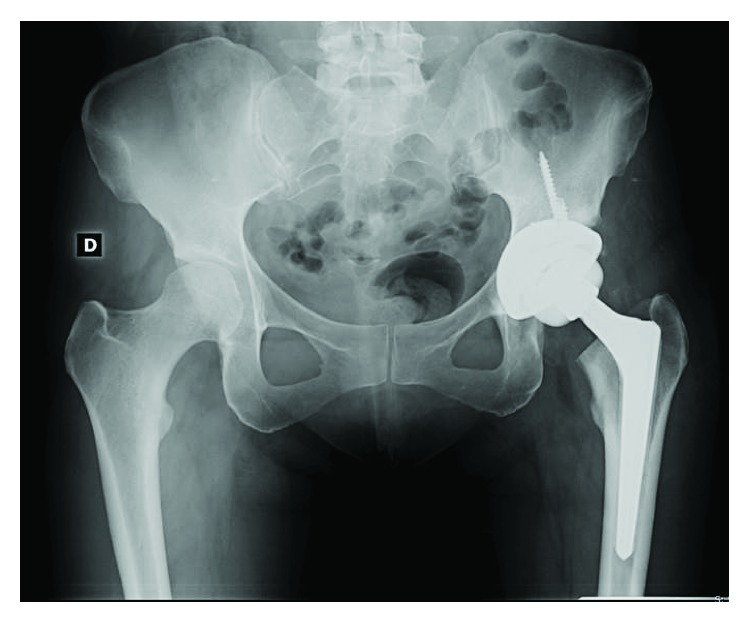
In an immediate post-RHA pelvis anteroposterior X-ray, an adequate position of the components is seen.

Then, the patient was repositioned in lateral decubitus, and a posterolateral hip approach was performed. The capsule was looking distend; it was punctured and then, a 30 cc fluid was obtained, similar to the liquid found in the retroperitoneum. After capsulotomy and femoral neck osteotomy, all arthroplasty components were removed, noticing an anterosuperior wall defect in the acetabulum. After complete pseudotumour resection, RHA was performed (Figure 5). The acetabular cup component was a cementless 56 mm Dynasty® (Wright Medical, Memphis, USA), and the femoral component was a high offset femoral cementless stem (Stellaris®, Mathys, Bettlach, Switzerland, no. 17). The bearing surface used was polyethylene-ceramic, being Dynasty® polyethylene liner, on the acetabulum and a 40 mm ceramic on the femoral head (BIOLOX®, Mathys, Bettlach, Switzerland). Post surgery, an anteroposterior pelvic radiograph was taken (Figure 6). After fourteen days, tissue cultures obtained at surgery were negative for infection.

**Figure 7:**
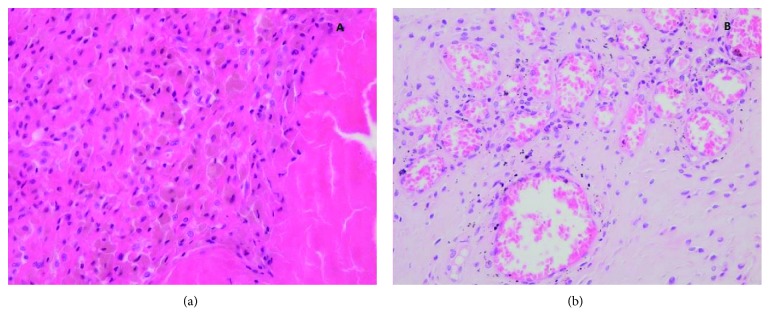
Histologic view of the soft tissue mass. (a) (40x) Hematoxylin and eosin (HE) shows an infiltrate of the macrophages with brown contents and detritus inside, near a necrotic area. (b) (100x) HE shows black particulates from the prosthetic material in a reparative tissue with fibroblast and neoformation of blood vessels.

Histopathological findings confirm the pseudotumour. The analyses reported an extensive aseptic inflammatory infiltrate of macrophages with detritus inside near a necrotic tissue area (Figure 7(a)). A PAS histochemical stain confirmed these findings. No signs of ALVAL were observed. Also, reparative tissue was found with black particulate material corresponding to the prosthetic material near the neoformation of blood vessels and fibroblast (Figure 7(b)).

**Figure 8:**
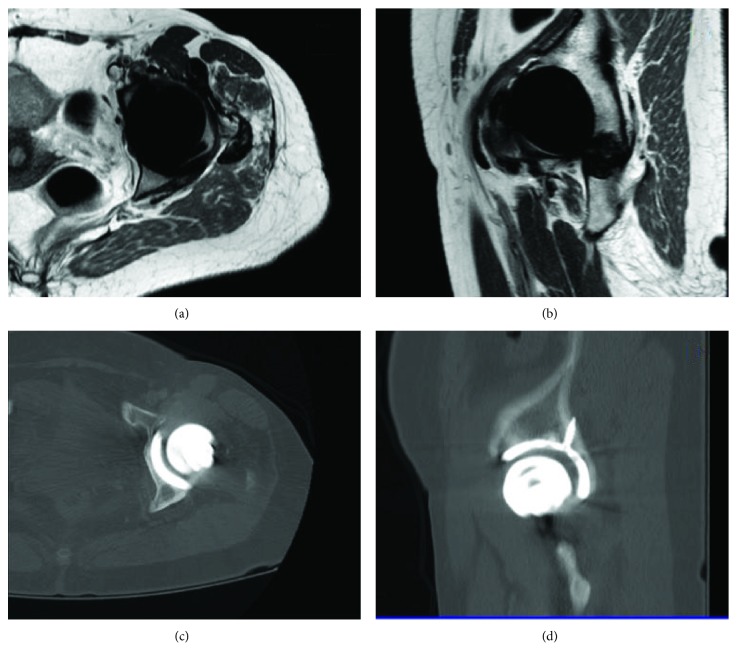
MRI (a) and (b) and CT (c) and (d) images taken five years post-RHA. There were no signs of pseudotumour after an extended follow-up.

No early- or late-surgery complication is reported. Since post surgery, the patient progressively recovers the hip's range of motion and normal gait. In the last follow-up, 60 months postoperatively, the patient is in excellent condition with no functional limitations and a full hip range of motion. Radiological exams, which included an annual MRI, showed no pseudotumour formation and no arthroplasty loosening. The MRI at five years of follow-up is shown in Figure 8.

## 3. Discussion

Pseudotumour is a rare complication of hip arthroplasty, and it is related to a metal-bearing surface [11]. The treatment planned in this case, which included complete resection of the pseudotumour and RHA, achieves excellent clinical outcomes, as the patient had no signs of pseudotumour after five years of follow-up.

It is well known that pseudotumour goes underdiagnosed; recent studies show that the prevalence of pseudotumour could be higher than previously reported [12]. In a cohort of 125 patients (143 hips, Birmingham® HRA), 28% had pseudotumour in the CT study. Most patients (72.5%) were asymptomatic. The most common symptoms described were a hip pain, a palpable mass, or paresthesia [13]. Another study showed a substantially higher incidence of pseudotumour formation in metal-on-metal THA, reaching 42 patients (39%) diagnosed with a pseudotumour, while 13 (12%) of these were symptomatic and were revised [9]. Moreover, delayed diagnosis drives to increase the size of the pseudotumour with more bone and soft tissue damage; therefore, there are more complications and poor clinical revision outcomes [14–16]. It is critical to remark that the longer the follow-up is, the higher the incidence of pseudotumour and its symptoms is.

Pseudotumour diagnosis is not easy as patients remain asymptomatic for a long time. As it is a well-known complication, screening with images must be performed on all prosthesis with a metal-bearing surface [17]. Moreover, we believe that the imaging study should include an MRI, which features high sensitivity and specificity for diagnosis, and a better definition of pseudotumour for diagnosis and operative planning [14, 18], especially in places where the measurement of metal ions in the blood is not available as it is in our case [19, 20].

Another issue that cannot be missed is that infection must be ruled out, as it is also a complication of THA and it transcends all prosthesis design [21]. For this, joint arthrocentesis and a synovial fluid analysis must be performed routinely. The liquid obtained must be sent to a traditional study and prolonged microbiological cultures for two weeks, according to international publications [22].

Regarding the aetiology of pseudotumours, current evidence attributes it to an adverse reaction to metal debris. Some studies indicate that this is due to a local inflammatory response to wear of the metal surface, which develops a granulomatous-like reaction proportional to the amount of the wear debris, and it correlates with the amount of metal ions in plasma [23, 24]. Beside the bearing surface, it has been reported as risk factor of hip dysplasia, malposition of prosthesis components, and larger size of the femoral head of the prosthesis hip dysplasia—all of them associated to excessive wear [8]. On the other hand, Willert et al. describe that pseudotumour is an aseptic lymphocyte-dominated vasculitis- (ALVAL-) associated lesion which is a delayed hypersensitivity reaction type and seems to be mildly related to the amount of wear debris. The most characteristic histological features of ALVAL were diffuse and perivascular infiltrates of lymphocytes and plasma cell infiltrates of eosinophilic granulocytes and necrosis. Only a few metal particles were detected [6, 25–27]. According to the recently published histopathological classification of joint implant-related pathology, this case corresponds to a “type 1: particle type,” in which the hallmark is the infiltrate of macrophages often with foamy feature and multinuclear giant cell, in which prosthesis wear can be detected [22].

Pseudotumour, as it has been established in previous paragraphs, is not an exclusive complication of RHA, but of those that use metal on metal-bearing surface. Even more, few cases have been reported supporting that pseudotumour could also be triggered by metal ion release from the head-neck taper junction in cases were no metal on metal-bearing surface was used [28]. The highest incidence of this complication in resurfacing prosthesis happens because they use only metal on metal-bearing surface [29]. A series with extended follow-ups of Birmingham® HRA had shown a significant rate of early failure, being the most frequent cause of a metal ion adverse reaction confirmed by histological analysis [2, 30]. Ollivere et al. report a series of 463 Birmingham® hip resurfacing [31], in which 9 of them had macroscopic and histological evidence of adverse reaction to metal debris. The main risk factors in this study were female gender, a small femoral component, a high abduction angle, and obesity. They do not recommend the use of Birmingham® HRA in these patients [31]. A more extensive series of 4226 hips with three types of HRA found 58 failures associated with adverse reaction to metal debris. The median ion concentrations in the failed group were significantly higher than those in the control group. Increased wear from the metal-on-metal-bearing surface was associated with an increased rate of failure secondary to an adverse reaction. Moreover, revision surgery seems to be useful to decrease the blood concentration of metal ions [32].

There are only case report evidences of the surgical treatment of this pathology [33–35]. Consensus exists to perform pseudotumour resection and RHA, mainly changing the metal-bearing surface; however, the amount of pseudotumour resection is not precise, and some authors prefer to resect what is around the hip, leaving the pseudotumor in more difficult areas to address such as the retroperitoneum [9, 36]. In our case, an aggressive tumour resection by a double approach was decided after considering the pseudotumour extension and neurological compromise. We believe that the complete resection of the pseudotumour is related to successful outcomes, and it is supported by some authors [32]. Until our last follow-up almost five years later, the patient had no clinical signs of pseudotumour, and annual MR imaging examinations had been negative. Tumour size and location matter, so it was technically challenging to remove it, as it was in close relation to the iliac and the femoral vessels. Retroperitoneum compromise is not often; Bosker et al. [9] described two cases where the pseudotumour extends into the abdominal space along the iliopsoas muscle. We believe that it is essential to perform surgery by a multidisciplinary team and complete resection of the pseudotumour [37]. A vascular surgeon was prepared if needed in our case, and the first step was performed by a surgeon, which was more familiar with the retroperitoneum approach.

Regarding RHA, a bone defect caused by the pseudotumour is an issue. In this case, fortunately, it was resolved by standard components, and it was not necessary to use complex revision arthroplasty components that add morbidity to the surgery. However, we consider that performing a RHA is not easy or risk exempt. In a study that compares the result of 53 revisions of different implants of Birmingham® hip resurfacing, the author concluded that the incidence of major complications after RHAs for pseudotumour (50%) was significantly higher than that of after RHAs for other causes (14%). The authors concluded that the outcome of revision for pseudotumour (16 cases) is poor, so consideration should be given to an early RHA before the tumour grows and a significant bone defect is present [10].

## 4. Conclusions

This case represents a rare but devastating complication related to metal-bearing surface arthroplasty, particularly to HRA. Currently, the evidence shows that the incidence of pseudotumours and the rate of revision for pseudotumours are higher than initially reported. So, the indication of a metal-bearing surface is questionable given the higher rate of early RHA compared to other bearing surfaces such as polyethylene or ceramic. The most remarkable of this case is that after complete pseudotumour resection and RHA, at five years of follow up, the patient achieved a complete range of motion, no pain, no limp, and no sign of recurrence in MRI. We encourage that for such cases, MRI for diagnosis should be performed, arthrocentesis to rule out infection, and complete resection of the pseudotumour and hip revision for treatment led by a multidisciplinary team. Also, we are in favour of not using the metal-bearing surface as the first option [38].
